# *In Situ* Monitoring of Intracellular Glucose and Glutamine in CHO Cell Culture

**DOI:** 10.1371/journal.pone.0034512

**Published:** 2012-04-03

**Authors:** Alireza Behjousiar, Cleo Kontoravdi, Karen M. Polizzi

**Affiliations:** 1 Division of Molecular Biosciences, Department of Life Sciences, Imperial College London, London, United Kingdom; 2 Centre for Synthetic Biology and Innovation, Imperial College London, London, United Kingdom; 3 Centre for Process Systems Engineering, Department of Chemical Engineering, Imperial College London, London, United Kingdom; Glasgow University, United Kingdom

## Abstract

The development of processes to produce biopharmaceuticals industrially is still largely empirical and relies on optimizing both medium formulation and cell line in a product-specific manner. Current small-scale (well plate-based) process development methods cannot provide sufficient sample volume for analysis, to obtain information on nutrient utilization which can be problematic when processes are scaled to industrial fermenters. We envision a platform where essential metabolites can be monitored non-invasively and in real time in an ultra-low volume assay in order to provide additional information on cellular metabolism in high throughput screens. Towards this end, we have developed a model system of Chinese Hamster Ovary cells stably expressing protein-based biosensors for glucose and glutamine. Herein, we demonstrate that these can accurately reflect changing intracellular metabolite concentrations *in vivo* during batch and fed-batch culture of CHO cells. The ability to monitor intracellular depletion of essential nutrients in high throughput will allow rapid development of improved bioprocesses.

## Introduction

Biopharmaceuticals, or protein-based drugs, are the fastest growing class of therapeutics with over 500 new molecules currently in development [1] annual sales of more than $90 billion USD in 2009 [2]. Approximately 70% of these molecules are glycoproteins [1]. The development of industrial processes for biopharmaceutical production is a slow and expensive process which involves the generation and maintenance of hundreds of cell lines which are assessed for the ability to produce high levels of high quality product at various scales and the optimisation of cellular growth medium to boost production levels and ensure consistent glycosylation patterns. Process development remains largely an empirical exercise as we have yet to establish a full understanding of the mechanistic link between process conditions/medium formulation, cellular metabolism, and product formation rates. Efforts at high throughput process development are currently hampered by a lack of information-rich assays on the small scale. Assessment of cell growth in small volumes by solely estimating confluence cannot be predictive of behaviour in industrial-scale fermenters [3]. Therefore, significant effort towards the development of miniaturized analytical devices to detect important metabolites in cell culture is currently underway. Many solutions now exist for detecting extracellular concentrations of metabolites using chemical (*e.g*. [4]), enzymatic (*e.g.* [5]), or binding protein-based assays (*e.g*. [6]).

A significant improvement to current practice would be the development of a system that offers the ability to monitor essential intracellular metabolite utilization rates *in situ*, non-destructively and in small volumes so this information could be incorporated earlier in process development. Förster resonance energy transfer (FRET) biosensors based on fluorescent proteins offer the possibility to measure metabolic functions *in vivo* and can form the basis of a platform for non-invasive cell culture monitoring. FRET biosensors have previously been used to monitor individual metabolites (*e.g*. [7]–[9]), track multiple targets [10], as well as to provide spatiotemporal information at the subcellular level [11].

In this paper, we report the development of a CHO cell culture system expressing protein-based biosensors for glucose and glutamine and present data for *in vitro* and *in vivo* calibration of their fluorescent signal. We further evaluate how other amino acids present intracellularly can compete with glutamine for the ligand binding site of the glutamine biosensor and, thus, interfere with the signal emitted by this biosensor and at which concentrations this adverse effect occurs. Finally, we assess how this system can be used in-process in batch and fed-batch cell cultures for obtaining data for intracellular concentrations of these two key nutrients. This constitutes a proof-of-principle for this *in situ* monitoring platform, which can be extended in the future to other vital metabolites. Such an approach would remove uncertainties arising from the choice of quenching and extraction methods, obviate the need for destructive sampling, and could provide real-time information for in-process decision making.

## Materials and Methods

### Cell line maintenance

CHO cells (CHO-S from Invitrogen, UK) were maintained in suspension in CD-CHO medium (which contains 36 mM glucose and 0 mM L-glutamine, Invitrogen, UK, Catalogue number: 10743-029) supplemented with 8 mM L-Glutamine and 10 ml/L 100× hypoxanthine/thymidine supplement (Invitrogen, UK, Catalogue number: 11067-030). The cells were subcultured every 3–4 days with a seeding density of 2×10^5^ cells mL^−1^ and were grown in 250 mL Erlenmeyer flasks in a working volume of 50 mL. They were kept in a cell incubator at 37°C, a humidified atmosphere of 5% CO_2_, on an orbital shaking platform rotating at 125 rpm.

### FRET sensor plasmid construction and transfection

The glucose FRET plasmid pRSET-FLIPglu600μΔ11Aphrodite (Addgene number 13569) was kindly supplied by Professor Wolf Frommer (Carnegie Institution for Science, Stanford University) and transformed directly into chemically competent *E coli* BL21(DE3) Gold cells (Agilent, UK) for protein production. FLIPglu600μΔ11Aphrodite is a fusion of the enhanced cyan fluorescent protein (ECFP) and the Aphrodite variant of the enhanced yellow fluorescent protein (EYFP) to the F16A mutant of the *E.coli* glucose/galactose periplasmic binding protein (*mgl*B). 11 amino acid residues were deleted in the linker region to optimise the FRET efficiency [12]. To construct the mammalian expression plasmid, the FLIPglu600μΔ11Aphrodite gene was PCR amplified from the original vector and cloned into the pCDNA4/TO vector (Invitrogen, UK) using the unique *Eco*RI and *Pst*I restriction sites.

The glutamine FRET construct in the pUTKan plant expression vector was kindly supplied by Dr Uwe Ludewig (Hohenheim University). The biosensor is based on an insertion of the yellow fluorescent protein citrine into the *E.coli* glutamine periplasmic binding protein (QBP) between amino acids 98 and 99 with the ECFP attached to the C-terminus. Several additional residues in the linking region were deleted in order to rigidify the linker structure. The amino acid substitution D157N has been made to the QBP to increase affinity for glutamine and several mutations have been incorporated to improve FRET efficiency [9]. The vector supplied was digested with *BamH*I and *Sal*I to release the insert which was subsequently cloned into the corresponding restriction sites in the vector pET41a in frame with the N-terminal purification tags. This was then transformed into competent BL21(DE3) Gold *E.coli* cells (Agilent, UK) for protein expression The mammalian expression vector was created by ligating the same insert into the pCDNA4/TO vector which had been digested with *BamH*I and *Xho*I, taking advantage of the compatible sticky ends produced by *Sal*I and *Xho*I.

The mammalian expression vectors containing the glucose and glutamine FRET sensors were transfected into CHO cells using the TransIT-Pro™ transfection kit (Mirus Bio) according to the manufacturer's protocol. Stable transfectants were selected using 400 µg/mL Zeocin (Invivogen).

### Production of sensors in *E. coli* and protein purification

Both FRET sensor proteins were expressed in BL21(DE3) Gold *E.coli* cells (Agilent, UK). For the glucose FRET sensor, a single colony of freshly transformed cells was inoculated into a 2 L plastic baffled flask (Nalgene), containing 500 mL LB broth supplemented with 50 µg mL^−1^ of ampicillin. The flask was incubated at room temperature in a Thermo scientific MaxQ horizontal shaking cabinet (225 rpm) for 2–3 days, in darkness as the flourophores are sensitive to light. After 2–3 days the cells were harvested by centrifugation for 15 minutes at 6000 rpm and 4°C. For the glutamine FRET sensor, a single colony was inoculated into a 2 L plastic baffled flask (Nalgene), containing 500 mL LB broth supplemented with 30 µg mL^−1^ of kanamycin. Cells were grown for one day at room temperature with shaking at 225 rpm, followed by the addition of IPTG to a final concentration of 0.2 mM. Cells were incubated for a further two days before harvesting by centrifugation as above.

For affinity chromatography, cell pellets were re-suspended in 5 mL ice cold lysis buffer (20 mM Tris-Cl, pH 8.0) and transferred to a 50 mL Falcon tube. The cells were lysed with a Sonics Vibra Cell sonicator using pulses of 15 seconds on, 15 seconds off for 3 minutes. Samples were then centrifuged for 1 hour at 5000 rpm to remove cell debris. The lysates were removed and 1 mL Ni-NTA resin (Qiagen) was added per 4 mL of protein lysate. The protein was bound to the resin for 1 ½ hours in a cold room, under gentle agitation. The resin beads were packed into a disposable plastic polypropylene column (Qiagen) and washed with 10 mL ice cold wash buffer (20 mM Tris-Cl, pH 8.0, 10 mM imidazole) twice at 4°C. The protein was eluted with 5×1 mL addition of ice cold elution buffer (20 mM Tris-Cl, pH 8.0, 500 mM imidazole). To obtain the highest concentrations of protein, the eluate was collected in 5 separate fractions. For the glucose sensor, the three most concentrated protein samples were injected into a Slide-a-Lyzer® dialysis cassette (Membrane Molecular weight cut-off 20.000 Da) and dialysed overnight against 50 mM sodium phosphate buffer pH 7.0 at 4°C to remove the imidazole. Dialysis caused the glutamine biosensor to precipitate, so this was used as eluted from the IMAC column. Protein concentrations were measured using a Nanodrop 1000 (Thermo Scientific) using an appropriate blanking solution. Proteins were stored overnight at 4°C before fluorescence measurements were taken.

### Batch and fed-batch CHO cell cultures

Batch overgrow cell cultures were maintained in duplicates for 11 days in 250 ml Erlenmeyer flasks with a working volume of 50 ml. Samples were removed at 24 h-intervals to determine the viable cell concentration by light microscopy using the trypan blue dye exclusion method. Additional samples for intracellular metabolite analysis were removed for metabolite extraction. These were handled on ice at all times to slow metabolism [13] and minimize metabolite loss. Samples were centrifuged at 800 rpm for 5 minutes at 4°C and the supernatant was removed. The pellet was re-suspended in 3 ml ice cold phosphate buffered saline (PBS) and centrifuged for 5 minutes at 800 rpm at 4°C as a wash step. The supernatant was again removed and the pellet was re-suspended in ice cold PBS as above. This was then sonicated on ice (Sonics, vibra cell) a total of 5 times for 3 minutes each at a pulse of 15 seconds on and 15 seconds off. Directly after sonication the cell extracts were frozen at −20°C. Fed batch experiments were conducted in duplicates as with the batch overgrow experiments in 250 mL of CD-CHO but with additional supplementation of glucose or glutamine on day 6 of the cell culture to bring the extracellular concentrations of these metabolites to 36 mM and 4 mM, respectively. Alongside these cultures, we maintained two control cultures that were fed with the same volume of pure water instead of nutrients.

### Metabolite assays

The intracellular concentrations of metabolites were determined enzymatically using cell extracts following sonication. To prevent destruction of metabolites during the measurement process, cell extracts were handled on ice at all times. Intracellular concentrations of glucose were determined using the Amplex ® Red Glucose Assay kit (Invitrogen, UK) according to the manufacturer's instructions. Briefly, this is a coupled enzyme assay which relies on glucose oxidase to convert the glucose to gluconolactone plus hydrogen peroxide and horseradish peroxidase to react the hydrogen peroxide with the Amplex ® Red reagent to produce a product which is both fluorescent and coloured. In our case, glucose concentrations were determined by monitoring an increase in absorbance at 560 nm compared to a standard curve of known concentrations. Intracellular concentrations of glutamine were determined using the EnzyChrom Glutamine Assay kit (Universal Biologics), a coupled glutaminase/glutamate dehydrogenase assay that produces a colourimetric response at 565 nm. Concentrations were determined by comparison to a standard curve with correction for the presence of intracellular glutamate as recommended in the manufacturer's instructions. The metabolite concentrations from the assays were converted to intracellular concentrations using a CHO cell diameter of 12 µm [14].

### Fluorescence measurements and FRET ratio calculations

All fluorescence measurements were made in a Tecan Infinite 200Pro fluorescence plate reader using an excitation wavelength of 430/35 nm and emission wavelengths of 465/35 nm (blue) and 520/10 nm (yellow). *In vitro* calibration curves were determined using purified protein at a final concentration of 0.3–0.5 mg/mL in phosphate buffered saline pH 7.2 using increasing concentrations of ligand. For glucose, concentrations ranged from 10 µM to 1 M, while for glutamine concentrations ranged from 50 µM to 80 mM. For each concentration, FRET ratios were calculated as the amount of yellow fluorescence detected divided by the amount of blue fluorescence detected after correction for the background fluorescence of a sample containing no protein. The FRET ratios obtained were compared with the published literature for previous use of the biosensors as a benchmark [9], [12]. Competition assays for the glutamine biosensor were conducted using purified protein and 1 mM glutamine supplemented with the maximum intracellular concentrations of each of the other amino acids reached at any point during batch cell culture from data reported in other studies (Table 1, [15]). *In vivo* fluorescence measurements were performed using cells sampled from a growing flask into a 6 well plate. Every 24 hours, a 2 mL sample was removed from the flask and pelleted by centrifugation at 800 rpm for 5 minutes at 4°C. The cell pellet was resuspended in 2 mL of ice cold PBS and pipetted into a 6 well plate. Blue and yellow fluorescence measurements were made immediately. A small part of the sample was then used for viable cell concentration determination using the trypan blue exclusion method and the rest of the sample sonicated as described in the metabolite assay section above. The FRET ratios were calculated as the amount of yellow fluorescence detected divided by the amount of blue fluorescence detected.

**Table 1:** Concentrations of amino acids used in the competition study.

Amino Acid	Range	Concentration Used	Reference
Alanine	9.4–10.7 mM	10 mM	Hansen and Emborg, 1994
Arginine	0–2.3 mM	3 mM	Hansen and Emborg, 1994
Asparginine	0.04–18.7 mM	20 mM	Hansen and Emborg, 1994
Aspartate	1.1–3.4 mM	4 mM	Hansen and Emborg, 1994
Cysteine^a^	0.5–5 mM	5 mM	CK, unpublished data
Glutamate	2.6–4.6 mM	5 mM	Hansen and Emborg, 1994
Glutamine	0.3–0.9 mM	1 mM	Hansen and Emborg, 1994
Glycine	4.4–8.4 mM	9 mM	Hansen and Emborg, 1994
Histidine	0.3–0.6 mM	0.5 mM	Hansen and Emborg, 1994
Isoleucine	0.5–0.8 mM	1 mM	Hansen and Emborg, 1994
Leucine	0.2–0.5 mM	0.5 mM	Hansen and Emborg, 1994
Lysine	0.2–0.6 mM	0.5 mM	Hansen and Emborg, 1994
Phenylalanine	0.6–0.8 mM	1 mM	Hansen and Emborg, 1994
Serine	0.5–1 mM	1 mM	Hansen and Emborg, 1994
Threonine	0.9–3.5 mM	4 mM	Hansen and Emborg, 1994
Tyrosine	0.03–0.5 mM	1 mM	Hansen and Emborg, 1994
Valine	0.6–1.2 mM	1 mM	Hansen and Emborg, 1994

a For cysteine, the maximum extracellular concentration was used as data on intracellular concentration was not reported and this metabolite was shown to produce a high FRET ratio in [9].

## Results and Discussion

### Biosensor selection

**Figure 1:**
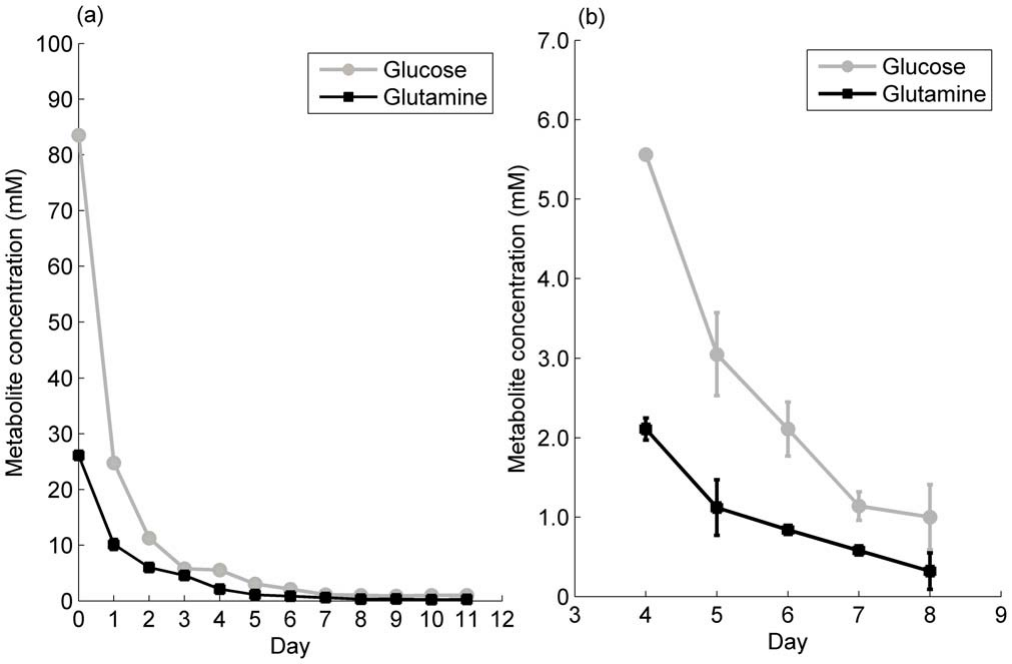
Intracellular metabolite concentration ranges during batch overgrow CHO-S culture. a) Glucose concentration measured by the Amplex Red ® glucose oxidase assay (gray circles) and glutamine concentration measured by a coupled glutaminase, glutamate dehydrogenase assay with correction for glutamate concentration (black squares) over the course of the batch overgrow culture. Error bars represent one standard deviation of the mean (n = 4 flasks with three enzymatic assay samples per flask) b) A closer view of the concentration of metabolites on days 4 to 8 (exponential to early stationary phase).

**Figure 2:**
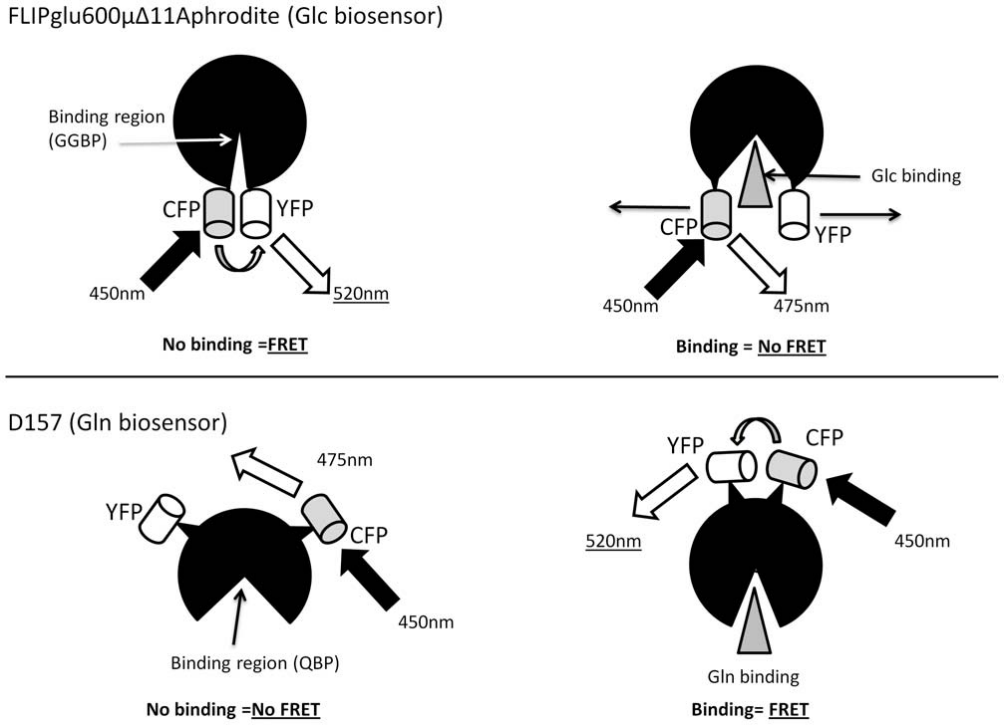
Diagrammatic representation of FRET sensor function. a) The glucose biosensor has maximum FRET efficiency in the absence of ligand. The binding of glucose causes a twist in the alignment of the fluorophores which causes the FRET ratio to decrease. b) The glutamine biosensor has maximum FRET efficiency in the presence of ligand which causes the binding domain to hinge closed, bringing the fluorophores into closer contact.

Intracellular metabolite data determined enzymatically from initial CHO cell cultures were used to determine the range of glucose and glutamine concentrations that occur over the lifetime of a batch overgrow experiment (Figures 1a and 1b) and, ultimately, to select biosensors with appropriate measurement ranges. Intracellular concentrations of glucose are very high in the first few days of cell culture, but steadily decrease over time, reaching a low of approximately 400 µM on days 5 and 6 of culture. This coincides with the peak in cell number (data not shown). During the stationary and decline phase, intracellular glucose concentrations increase again. Overall, with the exception of the early days of cell culture, average intracellular glucose concentrations range from 0.4 mM to 6 mM. In contrast, the levels of glutamine remain low and comparable across the lifetime of the cell culture, varying only between approximately 0.4 and 1 mM. Based on these concentration ranges, the biosensors FLIPglu600μΔ11Aphrodite [12] and the D157N variant of the QBP [9] were selected from the literature as having a linear range of measurement that corresponds with the intracellular metabolite concentrations measured. These two biosensors both work on the principle of FRET between fluorophores of the blue and yellow wavelength, but show opposite trends with respect to metabolite concentration (Figure 2). In the case of the glucose biosensor, binding of glucose causes a disruption of the fluorophore alignment, resulting in a decrease in FRET, whereas in the case of the glutamine biosensor, binding of glutamine causes the fluorophores to move into closer proximity, resulting in an increase in FRET.

### *In vitro* calibration

**Figure 3:**
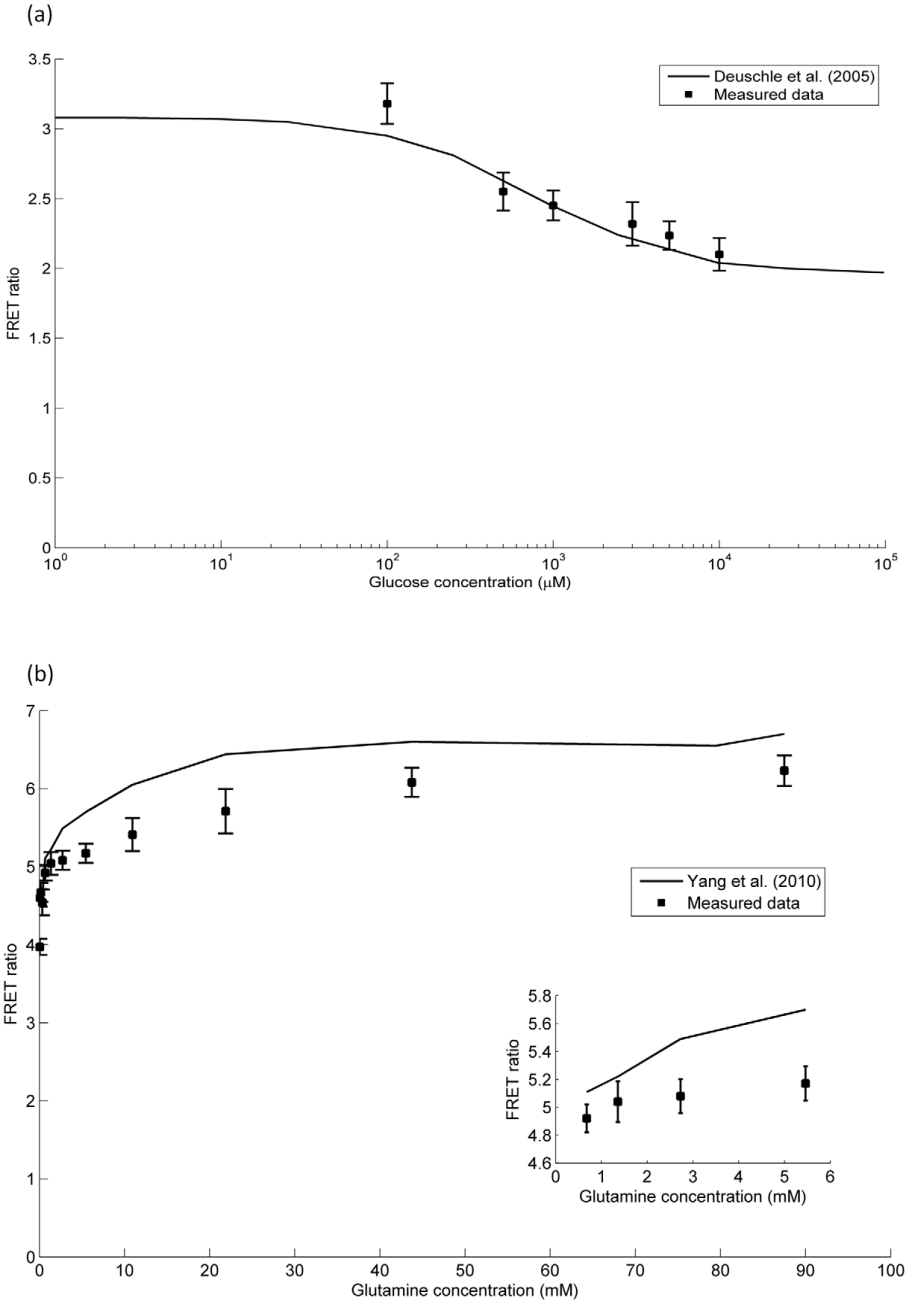
*In vitro* titration data in comparison with published results. a) Glucose biosensor measurements from Deuschle et al (2005) compared with titrations using purified protein extracted and measured as per the Materials and Methods section (n = 3 independent protein preparations, with 3 FRET ratio measurements per ligand concentration). b) Glutamine biosensor measurements from Yang et al (2010) compared with titrations titrations using purified protein extracted and measured as per the Materials and Methods section (n = 3 independent protein preparations, with 3 FRET ratio measurements per ligand concentration, error bars represent one standard deviation of the mean). Inset depicts a zoomed in view of the lower concentrations of glutamine.

In order to verify the linear range of measurement, the biosensors were overexpressed in *E coli* and the histidine tagged protein purified using immobilised metal affinity chromatography. Purified protein was then titrated against different concentrations of ligand and the results compared against published values as a benchmark (Figures 3a and b). Overall, our measured FRET ratios were in close agreement with those previously reported and the linear range of concentration measurement appears to be preserved. However, for the glutamine biosensor, the absolute values of our FRET ratios were consistently lower than those reported. Since the relationship remained linear, had the same midpoint, and appears to be directly downshifted from the values reported by Yang et al, this suggests a systematic difference arising from our experimental apparatus. Indeed, Yang *et al.* used an excitation filter of 433/12 nm and an emission filter of 475/12 nm for their ECFP measurements, which would have experienced less bleed through of the excitation wavelength than our filter set (430/35 nm, 465/35 nm). This is the most likely source of the discrepancy as our set-up would result in slightly higher blue fluorescence measurements, systematically decreasing the FRET ratio calculated.

### *In vivo* calibration

**Figure 4:**
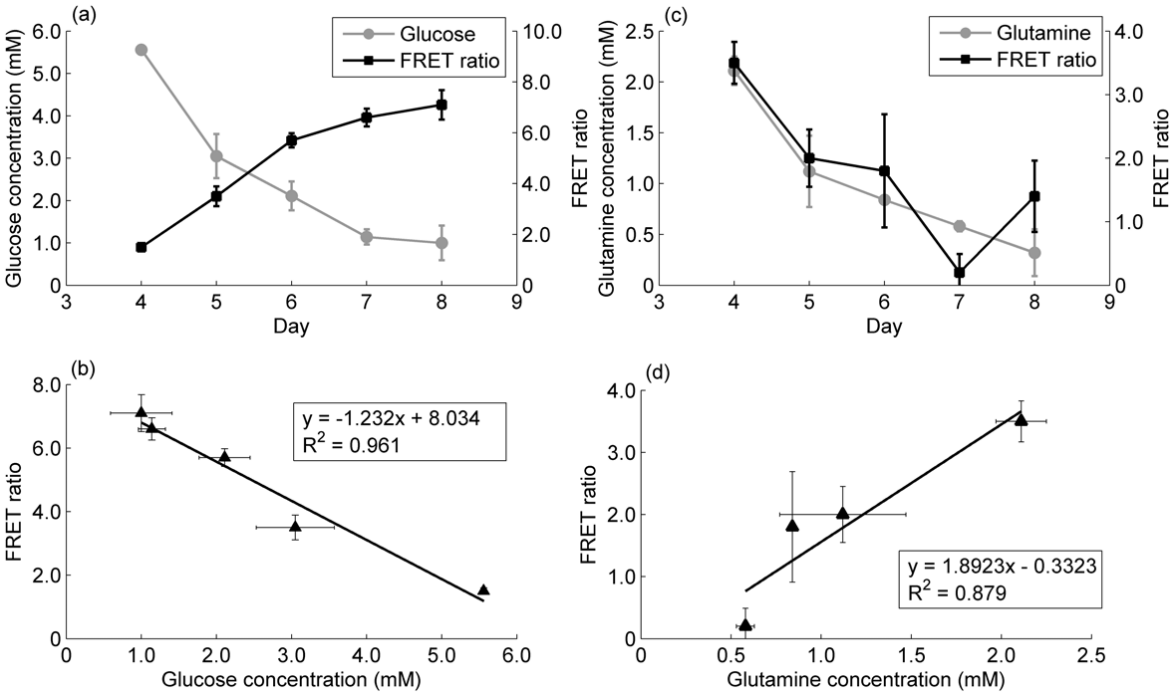
*In vivo* calibration curves. A comparison of the FRET measurements obtained from stable CHO-S cell lines constitutively expressing the biosensors and the corresponding measurements of intracellular concentrations from the same samples. a) Glucose biosensor measurements. Intracellular concentration as measured by the Amplex Red ® assay (gray circles) and corresponding FRET measurements (black squares). b) FRET versus concentration calibration curve for the glucose biosensor. The line represents the linear regression best fit for the data points. c) Glutamine biosensor measurements. Intracellular concentration as measured by the coupled glutaminase glutamate dehydrogenase assay (gray circles) and corresponding FRET measurements (black squares). d) FRET versus concentration calibration curve for the glutamine biosensor. The line represents the linear regression best fit for the data points. In all cases, n = 2 biological repeats with three measurements per cell line. Error bars represent one standard deviation of the mean.

FRET measurements *in vivo* are complicated by the fact that cell wall constituents and macromolecules can absorb and reflect light, leading to noise in measurements. In particular, this is a problem with lower wavelength light. Therefore, the *in vitro* calibration curves cannot be used directly to estimate the concentration of metabolites within the cells; an *in vivo* calibration must also be performed. Strangely, this step is often omitted in published work, but is vital for quantification. We transfected the mammalian expression plasmids into CHO cells and selected for stable integration of the plasmid. In order to calibrate the biosensor, we capitalised on the fact that intracellular concentrations of glucose and glutamine vary naturally with time. Thus, by sampling cells over the course of several days, we would be able to benchmark against different intracellular concentrations, at the same time ensuring that we were able to measure the range of metabolite concentrations which would be occurring naturally within our cell population of interest. Figure 4a shows the results of the *in vivo* calibration of the glucose biosensor with simultaneous measurements of the FRET ratio of intact cells and the intracellular glucose concentration (measured in cell extracts sampled from the same cultures at the same time as the FRET ratio was measured). FRET measurements before day 4 of the cell culture were subject to a high degree of variability due to low cell numbers, and after day 8 the presence of a significant number of lysed cells results in a high amount of light scattering (data not shown). Thus, the graph shows only the results for days 4 to 8. Figure 4b displays the same data as a calibration curve. The high R-squared value for the calibration curve indicates a high degree of correlation between the FRET measurements and the intracellular glucose concentration.

Figures 4c and 4d show the corresponding measurements for glutamine. In the case of the glutamine biosensor, the degree of fit is lower than that for the glucose biosensor, possibly due to interference by other amino acids as analysed in the section below. However, the calibration curve provides a means of determining the intracellular concentration range of glutamine with some degree of accuracy nonetheless. We note that the biosensor signal is best correlated with the intracellular concentrations between 1 and 7 mM for glucose and 0.5 and 2 mM for glutamine measured on days 4 to 8 of a batch cell culture. Although this is a limitation, these days correspond to the exponential, stationary, and early decline phases of cell culture, which are the most important to understand for bioprocess development. Additionally, other operational modes, such as fed-batch (see below) or continuous cultures would have a longer viable period of measurement, due to sustained high viability and cell concentration.

### Amino acid interference study

The *mgl*B protein upon which the glucose biosensor is based has a well known substrate specificity and has been demonstrated to bind glucose and galactose with approximately equal affinity [16] with very low affinity for other sugars [17]. Given the significantly low galactose uptake rates compared in industrial CHO cell culture even when it is the sole carbon source, *i.e.* in the absence of glucose [18], we felt that the glucose biosensor would not be subjected to interference by other metabolites in our experimental set up.

On the other hand, very little data exist on the ability of the glutamine binding protein to bind other amino acids. Previous work indicated that the glutamine biosensor was also able to bind to other amino acids and produce a FRET signal [9]. In particular, they noticed large positive FRET ratio changes upon addition of millimolar levels of arginine, cysteine, and histidine and negative FRET ratios produced in response to aspartate, glutamate and lysine, when adding individual amino acids to the purified protein. However, for use *in vivo* what is more relevant is whether physiogically relevant levels of amino acids *interfere* with the ability of glutamine to enter the ligand binding domain, *i.e.* whether the addition of amino acids at the levels normally encountered within CHO cells in culture will affect our ability to measure glutamine concentration. For example, in the presence of glutamine, do arginine and cysteine still bind, or is glutamine preferred? Will any of the other amino acids prevent glutamine entering the active site even if they themselves do not produce a FRET signal? In batch and fed-batch cell cultures amino acid levels vary across culture time as some amino acids supplemented in the medium are depleted and other amino acids are synthesised as a result of metabolic activity. If any of these interfere with the signal of the biosensor in response to glutamine, this would create difficulties in utilising the sensor as the interference would be time-dependent and thus very difficult to correct for.

**Figure 5:**
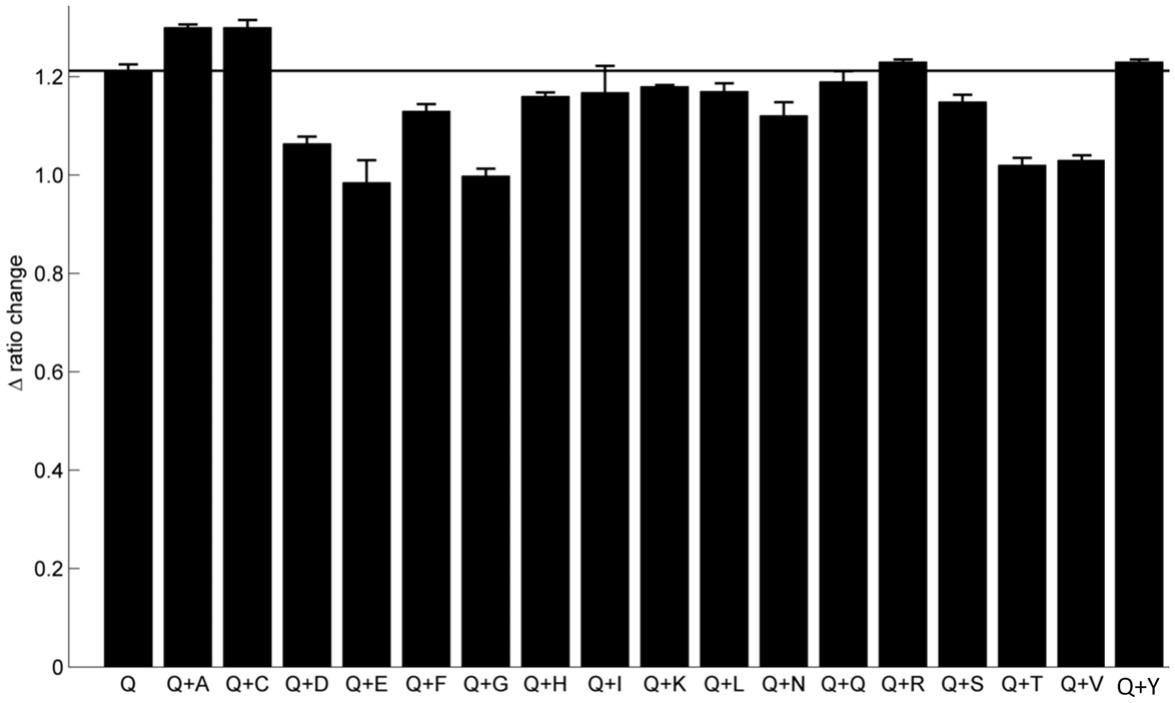
Assessment of the competition of other amino acids for the glutamine binding protein in the glutamine biosensor. The ability of the purified biosensor protein to accurately report the concentration of glutamine in the presence of other amino acids was measured using the maximum intracellular concentration of amino acid encountered during cell culture and 1 mM glutamine. Results are plotted as the change in FRET ratio between each condition and the FRET ratio of the purified biosensor with no ligand present. n = 3. Error bars represent one standard deviation of the mean.

Therefore, we tested whether the maximum concentration of amino acid encountered during the course of cell culture (as reported in Hansen and Emborg [15], Table 1) affected our ability to measure glutamine concentration accurately by adding individual amino acids to the purified protein in the presence of glutamine. Overall, most amino acids do not affect the ability of the biosensor to measure glutamine concentration accurately (Figure 5). In fact, the maximum difference in the measured FRET ratio change is approximately 20%, suggesting the level of interference is low. Interestingly, of the amino acids reported to bind to the biosensor and produce an increase in FRET in Yang et al, only cysteine produces a measurably higher change in FRET ratio when analysed in this context. Even asparginine, which is highly structurally similar to glutamine, does not produce a high level of interference. In addition to lower FRET ratios for aspartate and glutamate as reported, we also found decreased FRET ratio changes for glycine, threonine, valine, and tyrosine, indicating that these amino acids interfere with the ability of glutamine to bind to the active site of the protein when they are present. Glycine, threonine, and valine are relatively small amino acids and so could potentially enter the binding pocket of the biosensor and prevent glutamine access even if they themselves are not able to cause the conformational change necessary to produce a FRET ratio change. Taken together, these results suggest that the error in our ability to measure glutamine concentration is fairly low. This can be investigated further in situations where higher amino acid concentrations are anticipated due to the addition of complex feeds, even though a higher amino acid uptake rate may not necessarily result in higher intracellular concentrations but rather a higher metabolic activity and incorporation into proteins.

### Fed-batch culture results

**Figure 6:**
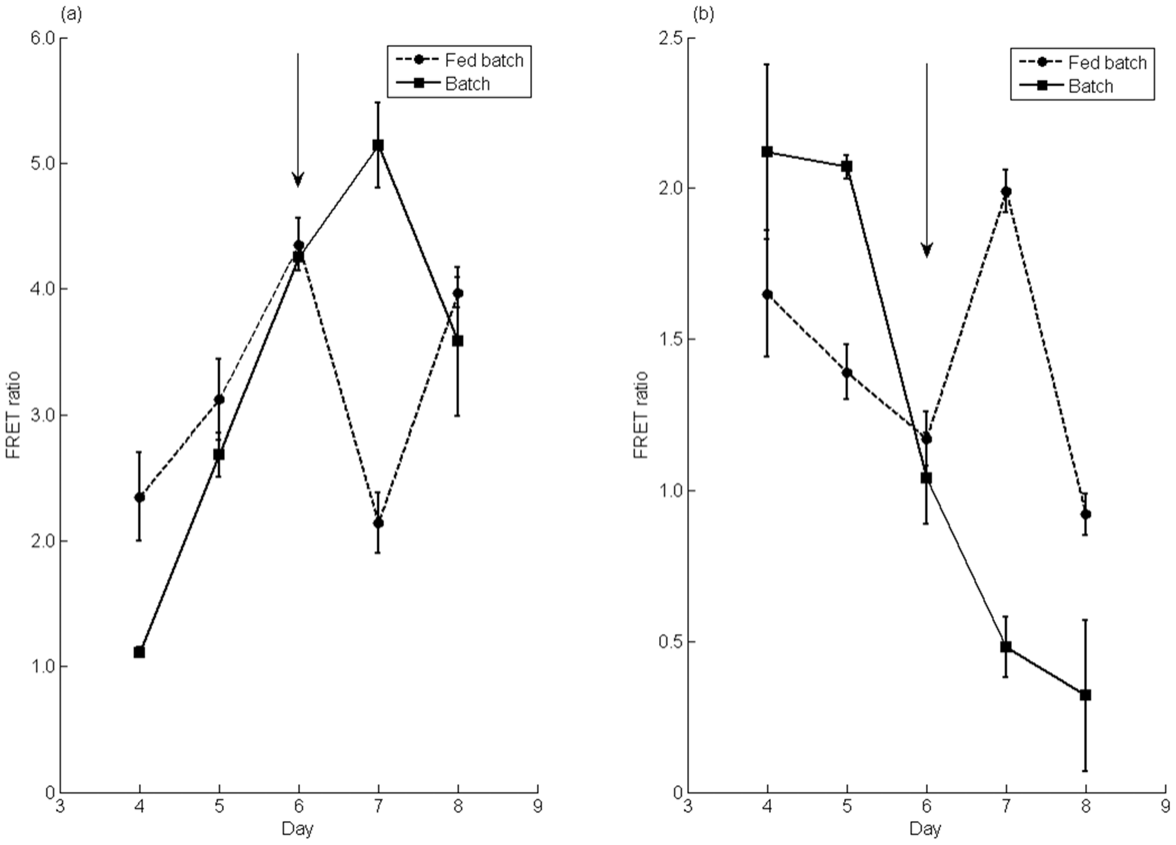
Fed batch culture of CHO-S cells and corresponding FRET measurements. a) Glucose fed-batch monitoring. Comparison of cells fed with glucose on day 6 (gray circles) and those given an equal volume of pure water (black squares). Arrow indicates addition of bolus feed to bring glucose concentration to 36 mM. b) Glutamine fed-batch monitoring. Comparison of cells fed with glutamine on day 6 (gray circles) and those given an equal volume of pure water (black squares). Arrow indicates addition of bolus feed to bring glutamine concentration to 4 mM. n = 3. Error bars represent one standard deviation of the mean.

In order to demonstrate that the two biosensors function in a bioprocessing context, we employed them for a study of fed-batch CHO cell cultures. Fed-batch culture is an operation mode whereby batch cultures are supplemented with additional nutrients later in cell culture in order to sustain cell viability and increase the culture lifetime and protein production. In this case, the cells were fed on day 6 of culture with a bolus addition of glucose and glutamine stock solution sufficient to bring the nutrient concentration up to36 mM for glucose and 4 mM for glutamine. Figures 6a and b compare the responses of the biosensor for cells that were fed versus those that were given a similar volume of pure water. The glucose biosensor has an inverse relationship between FRET and glucose concentration. Thus, one would expect that cells which had been fed glucose would show a significantly lower FRET ratio due to an increase in intracellular glucose concentration. Indeed, Figure 6a shows that this is the case: on day 7, cells that had been fed additional glucose have a FRET ratio that is less than one half of the ratio we obtained for the unfed control cultures. For the glutamine biosensor, increased glutamine concentration brings an increase in FRET, so addition of feed should result in an increased FRET ratio. Figure 6b shows that this is also the case, as the FRET ratio on day 7 is about 4 times higher in cells which were fed additional glutamine.

### Conclusions

We have demonstrated the utility of *in vivo* biosensors based on Förster Resonance Energy Transfer (FRET) for non-invasively monitoring the intracellular concentrations of primary metabolites in the batch and fed-batch cultivation of CHO cells. As a proof-of-principle, we chose to track two of the most important metabolites in mammalian bioprocessing: glucose, which acts as a primary energy source and carbon skeleton donor, and glutamine, which serves as an energy and nitrogen source. However, the methodology could be extended to monitoring other small molecules of interest and to other cell lines. In addition, while both of our biosensors use the blue and yellow fluorescent chromophores and so at present are mutually exclusive, strategies have been developed for simultaneously monitoring multiple targets using FRET pairs of different colours [10].

We selected the biosensors based on their ability to assess concentrations with a linear range of measurement that corresponds to the intracellular concentrations of glucose and glutamine encountered in cell culture and created stable cell lines that express the biosensor construct. Through simultaneous measurement of metabolite concentration and FRET ratio we were able to construct a calibration curve that can be used to accurately determine intracellular metabolite concentrations *in situ* without the need for quenching and metabolite extraction. We have further demonstrated that the acquired FRET ratio is subject to only minor alterations due to the presence of other metabolites, such as amino acids other than glutamine, and is therefore a reliable means for *in vivo* monitoring.

Importantly, the results demonstrate that the sensors would be useful in a bioprocessing context as we are able to get robust measurements of the intracellular concentrations of metabolites using cell sampling and a basic fluorescent plate reader, as opposed to requiring more sophisticated equipment such as a confocal microscope. This would allow for rapid data collection as well as open up the possibility of small scale screening assays for medium formulation and/or cell line engineering. Moreover, there is the possibility in the future to grow cells directly in the well plate and measure metabolite concentrations online, *in situ* in a low-volume, high throughput assay which would significantly speed up process development.
